# Regulatory mechanisms of plant rhizobacteria on plants to the adaptation of adverse agroclimatic variables

**DOI:** 10.3389/fpls.2024.1377793

**Published:** 2024-05-23

**Authors:** Krishan K. Verma, Abhishek Joshi, Xiu-Peng Song, Qiang Liang, Lin Xu, Hai-rong Huang, Kai-Chao Wu, Chandra Shekhar Seth, Jaya Arora, Yang-Rui Li

**Affiliations:** ^1^ Sugarcane Research Institute, Guangxi Academy of Agricultural Sciences/Key Laboratory of Sugarcane Biotechnology and Genetic Improvement (Guangxi), Ministry of Agriculture and Rural Affairs/Guangxi Key Laboratory of Sugarcane Genetic Improvement, Nanning, China; ^2^ Department of Botany, Mohanlal Sukhadia University, Udaipur, Rajasthan, India; ^3^ Department of Botany, University of Delhi, Delhi, India

**Keywords:** adverse agroclimatic conditions, physiological and omic aspects, plant responses, plant hormones, agricultural sustainability, rhizobacteria

## Abstract

The mutualistic plant rhizobacteria which improve plant development and productivity are known as plant growth-promoting rhizobacteria (PGPR). It is more significant due to their ability to help the plants in different ways. The main physiological responses, such as malondialdehyde, membrane stability index, relative leaf water content, photosynthetic leaf gas exchange, chlorophyll fluorescence efficiency of photosystem-II, and photosynthetic pigments are observed in plants during unfavorable environmental conditions. Plant rhizobacteria are one of the more crucial chemical messengers that mediate plant development in response to stressed conditions. The interaction of plant rhizobacteria with essential plant nutrition can enhance the agricultural sustainability of various plant genotypes or cultivars. Rhizobacterial inoculated plants induce biochemical variations resulting in increased stress resistance efficiency, defined as induced systemic resistance. Omic strategies revealed plant rhizobacteria inoculation caused the upregulation of stress-responsive genes—numerous recent approaches have been developed to protect plants from unfavorable environmental threats. The plant microbes and compounds they secrete constitute valuable biostimulants and play significant roles in regulating plant stress mechanisms. The present review summarized the recent developments in the functional characteristics and action mechanisms of plant rhizobacteria in sustaining the development and production of plants under unfavorable environmental conditions, with special attention on plant rhizobacteria-mediated physiological and molecular responses associated with stress-induced responses.

## Introduction

**Figure 1:**
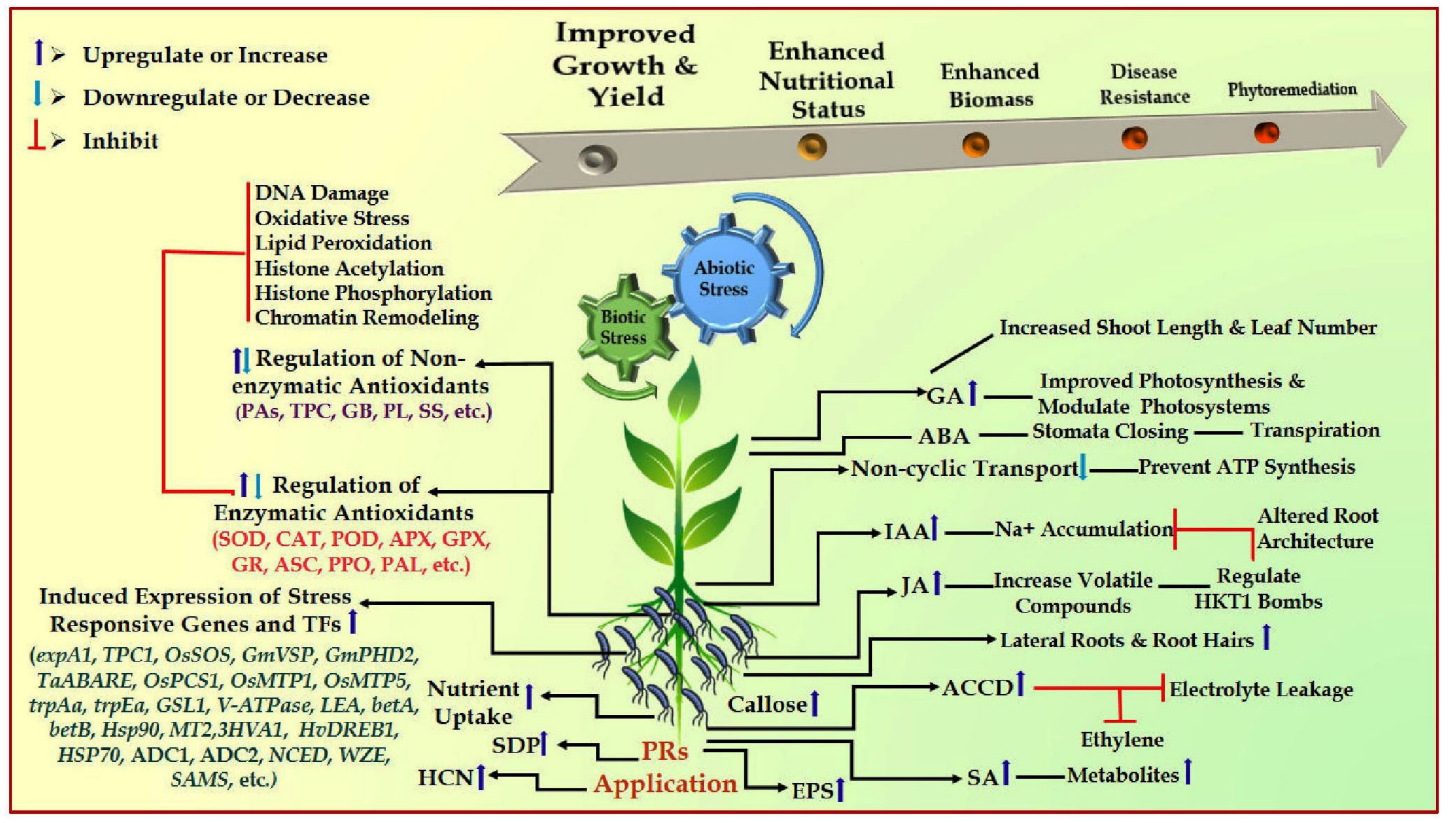
Schematic representation of PRs-mediated abiotic and biotic stress resistance mechanism in plants. ABA, abscisic acid; JA, jasmonic acid, GA, gibberellins, IAA, indole-3-acetic acid, SA, salicylic acid, EPS, exopolysaccharides, HCN, hydrogen cyanide; ACCD, 1-aminocyclopropane-1-carboxylate deaminase; SOD, superoxide dismutase; CAT, catalase; PAL, phenylalanine ammonia-lyase; APX, ascorbate peroxidase; POD, peroxidase; ASC, ascorbate; PPO, polyphenol oxidase; GPX, glutathione peroxidase; GR, glutathione reductase; Pas, polyamines; TPC, total phenolic content; PL, proline; SS, soluble sugar; HSPs, heat shock proteins; HKT—High-affinity K^+^ transporters; *expA1,* expansin*; TPC1,* calcium transporter; ADC1 and ADC2, putrescine synthesis; *OsPCS1, * phytochelatin synthase; *OsMTP1,* gene related to metal transport; *OsMTP5*, gene related to expulsion of excess metal*; trpAa*, and *trpEa,* genes related to tryptophan biosynthesis; *betA* and *betB*, genes related to betaine biosynthesis; *GmVSP* and *GmPHD2,* stress responsive genes; *GSL1,* gene related to cell wall synthesis; V-ATPase, Vacuolar-H^+^ -pyrophosphatase; LEA, late embryogenesis abundant; NCED, WZE and SAMS = transcription factors.

Plant rhizobacteria-mediated abiotic stress reduction occurs directly through hormone induction or indirectly via signaling in the host plant. The direct function in nitrogen fixation, phosphate solubilization, auxin, cytokinin, gibberellin, and abscisic acid production are all documented. It also makes it easier for necessary mineral elements to be absorbed from the rhizospheric soil along with the production of plant growth regulators. However, the indirect roles include the production of metabolites, siderophores, antibiotics, volatile HCN, etc. Some of the compounds that the microbes may produce include deaminase enzyme, microbiocidal enzyme, siderophores, plant hormones, and PO_4_-solubilizing enzyme (Gujral et al., 2013; Ekinci et al., 2014; Saleem et al., 2015; Kumari and Khanna, 2016; Moustaine et al., 2017). Plants have unique microbiota, and the microbial structure in the rhizosphere is influenced by the bacteria and plants’ production of signal molecules and the chemical composition of root exudates (Zhang et al., 2017; Jalmi and Sinha, 2022). Plant-growth regulators, phytohormones, and various secondary metabolites can be produced by PRs to stimulate plant development (Islam et al., 2014; Kaushal and Wani, 2016) (**Figure 1**).

The upregulated synthesis of metabolites, such as phytohormone, exopolysaccharides, siderophores, antioxidant enzymes, and volatile compounds, primarily minimizes plant resistance to environmental challenges. The production of phytohormones by rhizobacteria-inoculated plants, including cytokinins (CK), gibberellic acid (GA), indole-3-acetic acid (IAA), and abscisic acid (ABA) is employed during plant stressed conditions. 1-aminocyclopropane-1-carboxylate (ACC) deaminase plays a significant role in conferring stress resistance capacity to plants by downregulating the level of stress-induced ethylene level in plant roots system (Etesami and Maheshwari, 2018; Shahid et al., 2023). Plant-rhizobacteria downregulated the effects of abiotic stresses by modifying the expression of genes associated with the biosynthesis of hormones, i.e., *ACO* and *ACS* genes (ethylene biosynthesis), *MYC2* (Jasmonate), *PR1* (SA), genes encoding antioxidant enzymes, transcription factor *NAC1*, etc. (Tiwari et al., 2017) (**Tables 1**, **2**). Extensive field trials are required to investigate the interaction between the functional activities of signaling networks and their association. The interaction between PRs and plants based on various factors, such as root composition, strains of bacteria, and exudation patterns from their roots (Kumar et al., 2019). Numerous secondary metabolites and root exudates depend as chemo-attractants in the rhizosphere, attracting beneficial soil bacteria and inhibiting phytopathogens, thereby stimulating a delicate network of signaling between microbes and plants (Ullah et al., 2021; Joshi et al., 2022; Mellidou and Karamanoli, 2022; Joshi et al., 2023). The physiological and molecular responses activated in plants in response to stress resistance are regulated by various key genes with metabolic and regulatory roles. Research demonstrations focusing on plant gene expression following plant-rhizobacteria inoculation may help understand which can be an effective environmentally friendly approach to alleviate the adverse environmental variables (Ferrante et al., 2023; Verma et al., 2023). The formation of the enzyme ACC deaminase by rhizobacteria and reduction in ethylene level had been the main function for enhanced plant growth and resistance ability during different stresses (Bharti et al., 2014; Jalmi and Sinha, 2022).

**Table 1:** PR-mediated abiotic stress reduction in crop plants and their tolerance mechanism.

Stress condition	Plant	PR strains	PRs-mediated possible tolerance mechanism	Source
Cold	Tomato	*Streptomyces* sp. TOR3209	Upregulation of genes related to biosynthesis of abscisic acid (ABA), stress-related metabolism and photosynthesis	Ma et al., 2023
Cold	Maize	*Lysinibacillus fusiformis* YJ4*, L. sphaericus* YJ5	Upregulation of genes related to osmolytes, phenolic content, superoxide dismutase (SOD), catalase (CAT), phenylalanine ammonia-lyase (PAL), indole-3-acetic acid (IAA), and gibberellic acid (GA_3_)	Jha and Mohamed, 2023b
Cold	Wheat	*Bacillus* spp. CJCL2, *B. velezensis* FZB42	Downregulation of ABA and lipid peroxidation encoding genes *ABARE* and *4-HNE*, upregulation of gene related to Expansin *(expA1)*, Cytokinin *(CKX2)*, and Auxin *(ARF)*	Zubair et al., 2019
Drought	Barley	*Providencia rettgeri*	Increased production of IAA, siderophores (SDP), proline(PL), exopolysaccharides (EPS), and reduced level of malondialdehyde (MDA)	Ferioun et al., 2023
Drought	Chickpea	*Stenotrophomonas* sp. CV83	Upregulation of genes related to antioxidant enzymes SOD, POD, ascorbate peroxidise (APX), and lipoxygenase	Sharma et al., 2023
Drought	Maize	*Cronobacter* sp.Y501	Constrain ABA signaling, increase IAA biosynthesis, decrease MDA, SOD, CAT, peroxidase (POD) activity	Gao et al., 2023
Drought	Rice	*Pseudomonas putida* AKMP7	Polyamines (PAs)homeostasis through biosynthesis, back-conversion and catabolism of PAs	Nikhil et al., 2023
Drought	Soybean	*Bacillus pumilus* SH-9	Downregulation of ABA, upregulation of SOD, POD, APX, glutathione (GSH), EPS and SPD	Shaffique et al., 2023
Drought	Wheat	*Enterobacter bugandensis* WRS7	Overexpression of genes related to antioxidants (*CAT*, *APX*, *GPX*), osmolyte (*P5CS*, *P5CR*, *TPS1*), stress hormone (*NCED, WZE, SAMS, ACS1*) and *ACO* encoding proteins for ABA, ethylene, and calcium transporter (*TPC1*)	Arora and Jha, 2023
Heat	Tomato	*Bacillus safensis SCAL1*	Increased level of ACCD, EPS, IAA, gibberellic acid (GA_3_), kinetin, SOD, CAT, POD	Mukhtar et al., 2023
Heat	Maize	*Bacillus* spp. AH-08, AH-67, SH16 and *Pseudomonas* spp. SH-29	Upregulation of heat shock proteins (HSP1, *HSP18, HSP70, HSP101*), CAT, POD, and carotenoids	Ahmad et al., 2023
Heat	Mustered	*Bacillus aryabhattai* NSRSSS-1*, B. licheniformis* SSA 61, *Bacillus* sp. MRD-17	Increased production of IAA, GA, CAT, SOD, APX, phenolic content and reduction in PL, and soluble sugar (SS)	Kiruthika et al., 2023
Heat	Wheat	*Bacillus safensis*	Elicited expression of ADC1 and ADC2 linked to putrescine synthesis, modulated expressions of HSPs, upregulate redox enzymes and antioxidants associated with ascorbate (ASC)-GSH cycle, enhanced GB, SS, and phenols	Sarkar et al., 2021
Heavy metal	Barley	*Rhodospirillum* sp. JY3	Enhanced production of POX, CAT, SOD, GSH, ASC, polyphenols, phytochelatins, glutaredoxin, thioredoxin, peroxiredoxin	Alsiary et al., 2023
Heavy metal	Barley	*B. glycinifermentans* IS-2	Modulation of endogenous phytohormones and uptake of essential elements (K, P)	Belhassan et al., 2024
Heavy metal	Maize	*Agrococcus terreus* (MW 979614)	Augmented levels of antioxidant enzymes (SOD, POD), and nutrient uptake	Shahzad et al., 2023
Heavy metal	Maize	*Serratia* CP-13	Upregulate IAA, osmolytes (SS, PL), antioxidants and downregulate MDA, ABA, and Cd uptake	Tanwir et al., 2023
Heavy metal	Rice	*Serratia marcescens* DB1	Decreased expression of genes related to phytochelatin synthase (*OsPCS1)*,metal transport *(OsMTP1)*, expulsion of excess metal (*OsMTP5)*	Bhatta et al., 2023
Heavy metal	Tomato	*Serratia* sp. D23, *Sphingomonas* sp.	Upregulation of defense genes (*Hsp90*, *MT2* and *Nramp 3*)	Wei et al., 2022
Salt	Barley	*Siccibacter* sp. C2	Overexpression of *HVA1*, *HvDREB1, HvWRKY38*, *HvP5CS* genes	Sayahi et al., 2022
Salt	Chickpea	*Bacillus* sp. BSE01	Maintained levels of ACC, ABA and K^+^/Na^+^ ratio, enhanced production of, antioxidant enzyme, PL and decreased activity of NADPH oxidase	Basu et al., 2023
Salt	Lettuce	*Bacillus velezensis* JB0319	Induce SOD, POD activity and decreased MDA	Bai et al., 2023
Salt	Maize	*Pseudomonas* sp. MHR6	Induce production of EPS, reduce MDA and electrolyte leakage (EL)	Liu et al., 2022
Salt	Mustered	*Pseudomonas fluorescens*	Augmented production of glycine-betaine (GB), PL, SOD, CAT, APX and GR	Khan et al., 2023
Salt	Oat	*Bacillus* sp. LrM2	Induced production of ACCD, non–enzymatic antioxidants, ASC, GSH, dehydroascorbate	Zhang et al., 2023
Salt	Rice	*Pseudomonas promysalinigen* RL-WG26	Induce biosynthesis of tryptophan (*trpAa, trpB, trpC, trpD, trpEa*), IAA (*iaaM, iaaH*), betaine (*betA, betB, betT*) and inhibit ethylene biosynthesis (acdS) related transcripts	Ren et al., 2024
Salt	Rice	*Lysinibacillus fusiformis, L. sphaericus, Brevibacterium pityocampae*	Increased expression of JA,*OsNHX1*,*OsAPX1*, *OsPIN1, OsCATA* gene and reduced expression of ABA, salicylic acid (SA), and *OsSOS* gene	Asif et al., 2023
Salt	Soybean	*Streptomyces lasalocidi* JCM 3373	Induce expression of indole-3-carboxaldehyde (ICA1d), expression of stress-responsive genes (*GmVSP, GmPHD2*, *GmWRKY54)* and root growth related genes (*GmPIN1a, GmPIN2a, GmYUCCA5*, *GmYUCCA6)*	Lu et al., 2024
Salt	Tomato	*Bacillus halotolerans* Gb67*, B. subtilis* All3, *B. mojavensis* Gb7	Induced production of PAs, VCs, EPS and ACCD	Abdelkefi et al., 2024
Salt	Wheat	*Variovorax* sp. P1R9	Increased SOD, CAT activity and reduced thiobarbituric acid reactive substances (TBAR_S_)	Acuna et al., 2024
Salt	Wheat	*Nocardioides* sp.	Induce expression of ACCD, *TaABARE*, TaHAk1, hkt1, *CAT*, *MnSOD*, *POD*, *APX*, *GPX*, and *GR* gene transcripts	Meena et al., 2023

**Table 2:** PR-mediated biotic stress reduction in crop plants and their tolerance mechanism.

Stress condition	Crop	PR strains	PRs-mediated possible tolerance mechanism	Source
Net blotch fungus (*Drechslera teres*)	Barley	*Paraburkholderia phytofirmans* B25	Upregulation of genes related to cell wall synthesis (*GSL1,GSL3*, and downregulation of genes related to defense (*CAT2, AOC, PRB*), phenylpropanoid pathway (*PAL2*, *F3’H*), isovitexin, and lipid compounds	Backes et al., 2021
Wilt disease (*Fusarium oxysporum)*	Faba bean	*Bacillus velezensis*, *B. paramycoides*, *paramycoides*	Induced production of hydrogen cyanide (HCN), siderophores (SPD), indole-3-acetic acid (IAA), abscisic acid (ABA), benzyl, kinten, ziaten, and gibberellic acid (GA_3_)	El-Sersawy et al., 2021
Wilt disease (*Fusarium oxysporum*)	Maize	*Pseudomonas pseudoalcaligenes* (EU921258), *Bacillus pumilus* (EU921259)	Induce expression of β-1,3 glucanase genes, improved photosynthetic pigment, and cell membrane stability	Jha and Mohamed, 2023a
Wilt disease (*Fusarium oxysporum f.* sp. *pisi*	Pea	*Bacillus subtilis* (IS1)*, B. amyloliquificiens* (IS6), *B. fortis* (IS7)	Upregulation of total phenolic compounds and enzymes of phenylpropanoid pathway	Raza et al., 2024
Sheath blight disease (*Rhizoctonia solani*)	Rice	*Bacillus velezensis*, *B. megaterium, B. toyonensis*	Increased activity of polyphenol oxidase (PPO), superoxide dismutase (SOD), catalase(CAT)	Patil et al., 2024
Leaf stripe disease (*Burkholderia*)	Sorghum	*A. chroococcum* Beijerinck 1901 (MCC 2351), *B. megaterium* (MCC 2336), *P. fluorescens* (NAIMCC B-00,340)	Decreased levels of malondialdehyde (MDA), proline, CAT, SOD	Rizvi et al., 2024
Speck disease (*Pseudomonas syringae* pv. tomato)	Tomato	*Pseudomonas koreensis* 5*, Bacillus mycoides* 68*, B. mojavensis* 36*,B. simplex* 47	High levels of proline, POD, CAT	Yildiz et al., 2023
Wilt disease (*Ralstonia solanacearum*)	Tomato	*Pseudomonas* *fluorescens* Pf3, *Trichoderma* *longibrachiatum* UNS11	Increased activity of peroxidase (POX), phenylalanine ammonia-lyase (PAL), and PPO enzymes	Konappa et al., 2020
Spot blotch disease (*Bipolaris sorokiniana*)	Wheat	*Bacillus subtilis* BS87	Increased levels of nutrient solubilization, SPD, IAA, HCN and decrease levels of SOD, POD, PPO, MDA, PAL, proline	Chandra et al., 2024
Fungal pathogens (*Alternaria alternata*, *Rhizoctonia solani*, *F. oxysporum*, *Ustilaginoidea virens*)	Wheat	*Beijerinckia fluminensis* BFC-33	Increased levels of carotenoid, PAL, PPO, β-1,3 glucanase and reduce proline, thiobarbituric acid reactive substances (TBAR_S_) and electrolyte leakage	Al-Shwaiman et al., 2022

Eco-physiological and omic responses of plant rhizobacteria required more attention and extensive field research demonstrations to increase stress resistance efficiency. Hence, the present article focused on the interactions between plants and rhizobacteria and their impact on tolerance to adverse agroclimatic variables for agricultural sustainability in an eco-friendly environment.

## Impact of plant development, biomass, and productivity

Plant rhizobacteria (PRs) effectively improve plant morphological structures during adverse environmental conditions. Abiotic stresses, such as acidic and alkaline soil, insufficient water supply, low and high temperature, UV-radiation, soil flooding, and contaminated/toxic soil, affect agronomic, anatomical, cellular, and metabolic activities (Glick et al., 2007; Verma et al., 2020a, b). Higher levels of phytohormones, defense-related proteins and enzymes, antioxidants, and epoxypolysaccharides cause PGPR-induced resistance (Kaushal and Wani, 2016). It is accomplished by changing transcriptional and signaling processes, which lead to altered gene expression when PRs are present. Because PRs produce phytohormones that change root shape and improve root development, surface area, uptake, and accumulation of nutrients, plant productivity increases in the presence of PRs (Mellidou and Karamanoli, 2022). They can also increase total plant productivity by helping to induce ACC-deaminase activity in plants. The potential of PRs enhancing plant growth and development varies due to differences in their properties, such as ACC-deaminase activity, IAA generation, root colonization, P-solubilization, etc (Ghosh et al., 2018; Gupta and Pandey, 2019). The defense mechanisms of plants against unfavorable agroclimatic conditions depend on the variation in the development of roots (Khoshru et al., 2023). Different PGPR strains can enhance the overall root system by increasing the total number of root tips, surface area, and structure of the roots under stressful conditions (Brambilla et al., 2022). Lowering the ethylene content increases the plant’s capacity to withstand stress by facilitating improved nutrition and water uptake capacity (Chieb and Gachomo, 2023) (**Figure 1**).

When under stress, PRs also improve the uptake of water and nutrients. The absorption of nutrients and antioxidant activities are associated with stress management. By diminishing the negative consequences of saline soil, inoculation with *Klebsiella oxytoca* (Rs-5) containing ACC-deaminase boosted plant establishment and increased the absorption of key mineral nutrients (Yue et al., 2007; Zahir et al., 2012). In a similar way, *Pseudomonas* spp. inoculation increased the antioxidative enzymatic activities and promoted the growth of plants during unfavorable climatic conditions (Fu et al., 2010; Jalmi and Sinha, 2022) (**Table 1**).

According to Zahir et al. (2009) and Orozco-Mosqueda et al. (2020), rhizobacterial strains have been explored to have a substantial influence on the improvement of a variety of plants, including cereals, legumes, and vegetables cultivated under challenging conditions. They also enhanced the production of exopolysaccharides and ACC-deaminase activity. PRs enhance plant growth in polluted soil by downregulating the level of ethylene (Dell’Amico et al., 2008). PRs with 1-aminocyclopropane-1-carboxylic acid (ACC) deaminase activity may promote plant development during stress. Compared to uninoculated plants, the inoculated plants with PRs containing ACC-deaminase activity improved plant growth and yield considerably. *Pseudomonas* sp. and *Acinetobacter* sp. have increased IAA and ACC-deaminase production in saline soil and enhanced stress tolerance efficiency in barley and oats (Kang et al., 2019).

It can be indicated by the significantly increased levels of chlorophyll, total phenolics, flavonoids, soluble sugars, protein contents, and antioxidative enzymatic activities, as well as the higher expression of stress-related genes, that resulted from inoculating Cd-stressed with *Serratia marcescens* BM1 in *Glycine max* L. plants. *Phaseolus vulgaris* subjected to the rhizobacterial consortia experienced reduced stress caused by salinity and improved overall plant growth and photosynthetic pigments (Gupta and Pandey, 2019). In tomato plants, *Streptomyces* sp. has been shown to reduce stress and promote growth (Palaniyandi et al., 2014). It has been observed that *Burkholderia phytofirmans* helps plants under drought stress (Naveed et al., 2014). They generate exopolysaccharides (EPS) during water-deficit conditions, enhancing seed germination and growth. Of all the strains, *Pseudomonas fluorescens* has the highest capacity to produce EPS and ACC deaminase. The saline rice field was employed by Sultana et al. (2020) to isolate rhizobacterial strains, which they found to enhance stomatal conductance, transpiration, and photosynthetic CO_2_ assimilation rate, all of which contributed to increased crop yield, fruit and grains quality. According to the latest research, *Azospirillum brasilense* Sp245 increased *Arabidopsis thaliana* growth, suggesting that MAMPs produced from plant-rhizobacteria are essential for plant cultivation (Méndez-Gómez et al., 2021) (**Tables 1**, **2**).

## Photosynthetic leaf gas exchange and chlorophyll fluorescence efficiency

Plant-rhizobacteria enhance inoculated plants’ photosynthetic response and leaf gas exchange capability during stress (Verma et al., 2020b; Jalmi and Sinha, 2022). By modifying the photosynthetic characteristics, osmolytes production, antioxidant machinery, and expression of stress-related genes, inoculating soybean plants with *Serratia marcescens* BM1 (PR) provides Cd tolerance to plants (El-Esawi et al., 2020). Under salt stress, *Bacillus amyloliquefaciens* SQR9 has demonstrated higher efficiency in photosynthesis and overexpression of the RBCS and RBCL genes in *Zea mays* plants (Chen et al., 2016). During bacterial strain inoculation, *Arabidopsis helleri* showed elevated photosynthesis and proteins associated with abiotic stress (Khan et al., 2021).

Enhanced photosynthetic pigments and the expression of important genes (*RBCS* and *RBCL*) regulating RUBISCO activities during stress condition (Sherin et al., 2022; Amaral et al., 2023). By modulating ion homeostasis, redox potential, photosynthetic CO_2_ assimilation rate, and the expression of stress-related genes, maize plants inoculated with *Serratia liquefaciens* KM4 revealed enhanced growth and stress tolerance (El-Esawi et al., 2018). Reduced phenol, flavonoid, and leaf relative water content and photosynthetic responses in maize plants have resulted from salinity stress, which also decreased root damage and water uptake. However, inoculating maize under salt stress with *Serratia liquefaciens* KM4 enhanced LRWC, photosynthetic characteristics, and the biosynthesis pathways of phenols and flavonoids, enhancing plant stress tolerance efficiency. In comparison to uninoculated plants, rhizobacteria-inoculated maize and white clover have demonstrated enhanced photosynthesis, soluble proteins, sugars, and enzymatic activities following inoculation with HAS31 rhizobacteria (Han et al., 2014) (**Figure 1**; **Table 1**).

## Uptake and accumulation of mineral nutrients and water balance

By altering the solubility and absorption of nutrients, PRs improve the bioavailability of nutrients in plants under abiotic factors. Through N_2_-fixation, mobilization, and the promotion of N_2_-fixers through their secretions, several rhizobacteria can reduce the volume of nitrogen (N_2_) supplementation required for plant growth (Shah et al., 2022; Khoso et al., 2024). Additionally, they change the shape and surface area of the roots, improving nitrogen bioavailability (Olenska et al., 2020). Elevating ammonium transporters’ expression improves nutritional absorption during stresses (Calvo et al., 2019). According to Gomez-Godínez et al. (2023), phosphorus (P) solubilizing PRs, such as *Azotobacter, Bacillus, Burkholderia, Erwinia, Pseudomonas, Serratia*, and *Rhizobium*, generate organic acids that chelate P-bound cations and make it available to plant roots. Furthermore, under Fe-deficient conditions, PRs assist in acquiring iron (Fe) by generating siderophores, which are low molecular weight organic molecules (Mohanty et al., 2021). Reducing metal ion availability and decreasing metal uptake, siderophores that generate PRs enhance plants’ survival under heavy metal stress (Dimkpa et al., 2009; Kumar et al., 2021) (**Tables 1**, **2**).

*Ocimum basilicum* L. has demonstrated the ability of PRs to enhance nutrient absorption and downregulate abiotic stresses (Rakshapal et al., 2013). Under salinity stress, PRs, such as *Pseudomonas* sp. and *Azospirillum* sp., increase nutrient availability, improving plant growth, biomass, and productivity (Noorieh et al., 2013). The application of rhizobial inoculants has been observed to trigger delayed senescence, as evidenced by higher potassium (K) ion levels and lower ethylene and cytokinin production. In plants with a higher K^+^/Na^+^ ratio, PRs boost the absorption of K^+^ ions by synthesizing *AtHKT1*, a high-affinity ion channel that promotes stress tolerance (Mahmud et al., 2021) (**Figure 1**).

## Biosynthesis of plant hormones and compatible solutes

Along with metabolites and signaling molecules, the majority of rhizobacteria produce phytohormones (Ahmad et al., 2022; Shah et al., 2022). Among these include gibberellic acid, cytokinins, indole acetic acid (IAA), and abscisic acid (ABA) (Tariq et al., 2023). IAA is produced by 80% of soil microorganisms, including *Pseudomonas* sp., *Bacillus* sp., *Burkholderia* sp., and *Rhizobium* sp (Khan et al., 2021). It has been shown that IAA-producing rhizobacteria stimulate crop production and plant growth when exposed to abiotic stress (Mellidou and Karamanoli, 2022). Numerous IAA-producing rhizobacteria increase root biomass, length, and surface area, which improves nutrient accumulation, uptake, and plant growth (Fasusi et al., 2023). Increased IAA levels also foster lateral roots’ growth, minerals’ absorption, and root exudates’ formation. It is well known that some PRs, including *Arthrobacter, Azotobacter, Bacillus, Pseudomonas*, and *Pantoea*, synthesize cytokinins that enhance nutrient availability as well as plant tolerance responses (Shah et al., 2022) (**Table 1**).

According to dos Santos et al. (2020), gibberellin-releasing PRs such as *Azospirillum, Shingomonas, Bacillus amyloliquafaciens*, and *Bacillus pumilus* can also promote plant growth and yield. Regulation of abscisic acid also played a significant role in stress resistance capacity influenced by rhizobacteria (Herrera-Medina et al., 2007). When pepper (*Capsicum annum*) is inoculated with *Serratia nematodiphila* (that produces gibberellin), the plant expands more under low-temperature stress, releases more GA_4_ and ABA, and lower salicylate and jasmonate activities (Kang et al., 2015).

The plant and bacterial species may impact the mechanism of ABA-mediated tolerance to stressful conditions. Under abiotic stress, specific PRs (strains of *Rhizobium* spp., *B. pumilus, B. lycheniformis, Achromobacter xylosoxidans*, and *Azospirillium brasiliense*) serve as ABA-stimulators or ABA-producers (Salomon et al., 2014; Egamberdieva et al., 2017). It can assist plants minimize water loss by activating Ca^+2^ channels that cause stomatal closure (Goswami and Deka, 2020; Grover et al., 2021). Greater ABA biosynthesis has been observed in *Arabidopsis* plants inoculated with the spermidine-producing *B. megaterium* strain (Zhou et al., 2016). By upregulating the gene expression that regulates ABA production, the rhizobacteria inoculation of rice with *Pseudomonas fluorescens* enhanced the plant’s resistance to stress. The upregulation of *TaWRKY* and *TaMYB* expression in ABA-signaling cascades has also been observed. It has also been suggested that specific rhizobacteria can use ABA as a carbon and energy source, limiting ABA uptake throughout the plant organs. These results indicated the changes in ABA-mediated signaling pathways as a means by which inoculated plants can survive abiotic challenges (Olenska et al., 2020; Mellidou and Karamanoli, 2022) (**Figure 1**).

It has also been demonstrated that using rhizobacteria minimizes the negative effects of ethylene generated under abiotic stress circumstances (Grichko and Glick, 2001; Nadeem et al., 2007; Zahir et al., 2008). Under abiotic stresses, rhizobacteria-inoculated plants have been demonstrated to modify ethylene biosynthesis-related gene expression (Lephatsi et al., 2021; Verma et al., 2021; Fadiji et al., 2022). Plants can be spared the toxicity of ethylene through the presence of rhizobacteria that contain ACC deaminase, which can hydrolyze ACC, the precursor of ET (Mellidou and Karamanoli, 2022).

The impact of *Paenibacillus lentimorbus* B-30488 inoculation on the reduction of abiotic stress in *Arabidopsis thaliana*, as well as by modifications in plant hormones and RSA-related gene expression. According to Khoshru et al. (2023), specific PRs also generate polyamines, which enhance root architecture and promote stomatal conductance and photosynthesis. The microbial community in the rhizosphere is mainly influenced by the exudates produced by plant roots, such as organic acids, mucilage, carbohydrates, sugars, and proteins, which also confer tolerance to inoculated rhizobacterial plants (Backer et al., 2018). Under abiotic stress, *Azospirillum* sp. has been demonstrated to accumulate appropriate solutes such as glutamate, proline, glycine, betaine, and trehalose (Saleena et al., 2002). *Phaenibacillus polymyxa* has been shown to possess the drought-responsive gene ERD15 (Timmusk and Wagner, 1999). Conjugated phytohormones and flavonoids in root tissue can be extracted or hydrolyzed by *Azospirillum*, releasing them in their active forms (Spaepen et al., 2007; Dardanelli et al., 2008; Saikia et al., 2010; Fahad et al., 2015).

The mechanisms of photosynthetic activity, hydraulic conductance, osmotic accumulation, and sequestering toxic ions are associated with rhizobacteria-stimulated resilience to stress (**Figure 1**). Groundnut inoculated with *Bradyrhizobium* under drought conditions demonstrated stress resistance due to amino acids produced from the nitrogenase to catalyzed the conversion of atmospheric nitrogen (N_2_) to ammonia (NH_3_) ions (Delfini et al., 2010; Enebe and Babalola, 2018). Furthermore, nitrogenase assists the supply of nitrogen to inoculated legumes, and these plants have been shown to produce more leaves due to more root nodules (Ferreira et al., 2011). To avoid desiccation, lower toxicity, and promote root growth, PRs also generate polysaccharides (Arora et al., 2010). A vital aspect of stress mitigation under environmental stress at the plant rhizosphere consists of forming biofilm and exopolysaccharide. One fascinating strategy PRs employ to mitigate the impacts of heat stress in plants involves the induction of osmoprotectants and heat shock proteins (HSPs) (Enebe and Babalola, 2018). Under stressful conditions, pepper plants treated with gibberellin-producing rhizobacteria showed a reduction in the level of salicylate and jasmonate. When the bacteria *Burkholderia phytofirmans* occurs, tomato plants produce more phenolics, proline, and starch under stress (Issa et al., 2018).

In plants under abiotic stress, PRs also improve proline synthesis. *Arthrobacter, Bacillus*, and *Burkholderia* are the main rhizobacteria that synthesize proline. Better stress tolerance in rhizobacteria-inoculated plants is mostly due to increased dissolved sugar levels and solute storage. Other potential strategies to reduce oxidative stress include stabilizing membranes, protein–protein complexes, and osmolytes, such as proline, glycine betaine, amino acids, and total sugars (Chieb and Gachomo, 2023).

## Influence of enzymatic, non-enzymatic, and lignin biosynthesis

The synthesis of the enzyme ACC deaminase is a well-known mechanism for rhizobacteria-led abiotic stress tolerance (Etesami et al., 2015; Gupta and Pandey, 2019). By lowering ABA levels, plants inoculated with ACC-producing PRs expand more rapidly; the growth hormones regulate the synthesis of secondary metabolites (Kang et al., 2019). By promoting the activity of antioxidant enzymes (SOD, APX, and CAT) and upregulating the genes involved in the ROS pathway, it enhanced stress tolerance (Habib et al., 2016). Because ethylene causes stress-induced H_2_O_2_ accumulation and apoptosis induction, ACC deaminase-producing PRs provide plants resistance against abiotic stress by lowering ethylene synthesis. It has been observed that inoculating different crops under stress with strains that include ACC-deaminase enhances plant development (Li et al., 2017; Singh and Jha, 2017; Namwongsa et al., 2019; Danish et al., 2020; Mellidou et al., 2021; Mellidou and Karamanoli, 2022).

Plant-to-microbe communication also occurs by an array of non-hormonal signaling molecules. Microbes produce volatile compounds (VOCs), signaling molecules that control plant growth and modify soil and plant health in response to stress (Ullah et al., 2021). Moreover, plants tolerate heavy metal stress due to rhizobacteria-releasing extracellular polymeric substances (EPS), which primarily help by lowering the metals’ bioavailability in the soil (Mishra et al., 2017). Some species of *Bacillus, Azotobacter, Burkholderia, Enterobacter*, and *Pseudomonas* can reprogram plants’ redox states, increasing their tolerance to environmental stresses. During stress, the overproduction of reactive oxygen species (ROS) changes redox states and causes DNA damage, proteins, and membrane fluidity, ultimately resulting in cell death. However, plants inoculated with PRs defended against abiotic stressors by activating their defense mechanisms. Antioxidant enzyme activity enhanced in an array of growth-promoting rhizobacterial species to assist them in combatting oxidative stress (Mitra et al., 2021; Mellidou and Karamanoli, 2022) (**Figure 1**; **Tables 1**, **2**).

Additionally, rhizobacteria are essential in reducing oxidative damage caused by various stressors, including heavy metals, water deficit, low and high temperatures, salt, and water scarcity. By lowering ROS levels in plant roots, rhizobacteria-induced antioxidant enzymes assist in reducing the stressors that plants experience in the environment. Additionally, they accelerate the growth rate in response to abiotic stressors by promoting the generation of antioxidant enzymes. Better stress tolerance in inoculated plants may be due to increased activities of antioxidant enzymes, such as catalase (CAT), ascorbate peroxidase (APX), or glutathione peroxidase (GPX) (Mellidou et al., 2021; Swain et al., 2021; Fadiji et al., 2022). Ascorbate peroxidase increased when tomato seedlings were inoculated with *Enterobacter* and subjected to abiotic stress. Gladiolus plants treated with rhizobacteria revealed increased levels of CAT and SOD activities as compared to their control group (**Figure 1**).

Tomato seedlings inoculated with *P. oryzihabitans* AXSa06 (having ACC deaminase) experienced mild oxidative stress and enhanced lipid peroxidation to trigger the antioxidant machinery (Mellidou et al., 2021). Under abiotic stress, tomato plants inoculated with a strain of *Sphingomonas* sp. revealed reduced lipid peroxidation, increased glutathione levels, and antioxidant enzyme activities (Halo et al., 2015; Mellidou and Karamanoli, 2022). In contrast, rhizobacteria inoculation has been demonstrated in additional studies to decrease the production of ROS-scavenging or stress-responsive enzymes that are important for plant protection in stressful environments (Gupta and Pandey, 2019; Goswami and Deka, 2020; Song et al., 2021; Verma et al., 2022a, b, c). The generation of defensive enzymes like chitinase and glucanase to the rhizobacteria stress-tolerance mechanism (García-Fraile et al., 2015). *Glycine max* plants inoculated with *Bacillus firmus* SW5 exhibit stress tolerance through alterations in root ultrastructure, antioxidant levels, and stress-related gene expression (El-Esawi et al., 2020). The production of oxalic acid, gluconic acid, and citric acid by stressed rhizobacteria plays a crucial role in the mobilization of heavy metals. Biofilm-forming rhizobacteria were inoculated into *Spartina densiflora* plants, resulting in increased levels of SOD, CAT, and APOX activities as well as a decrease in the induced oxidative stress index (OSI) (Perez et al., 2019; Khan et al., 2021; Bhat et al., 2022).

In *Cicer arietinum* plants, *Pseudomonas putida* MTCC5279 has been shown to reduce stress by enhanced ROS scavenging ability, modulation of membrane integrity, and accumulation of osmolyte (proline, glycine, betaine). These findings have also been validated by differential expression of genes involved in dehydration-responsive element binding, transcription factors expressed under abiotic stress, salicylic acid, jasmonate, transcription activation, SOD, CAT, APX, and GST (Tiwari et al., 2016; Chieb and Gachomo, 2023). In *Abelmoschus esculentus* plants, the presence of ACC-producing PRs was associated with increased activities of antioxidant enzymes (SOD, APX, and CAT) and up-regulated genes of the ROS pathways (CAT, APX, GR, and DHAR) (Habib et al., 2016). These pathways have also been linked to enhanced POD/CAT activity, decreased cell death, and increased glutathione levels for ROS scavenging. When *Dietzia natronolimnaea* was inoculated into wheat (*Triticum aestivum*), it was observed that the ABA-signaling cascade genes, ion transporters, salt overly sensitive (SOS) pathway, and antioxidant enzymes upregulated (Bharti et al., 2016) (**Figure 1**).

## Conclusion and future prospects

Adverse environmental variables severely affect crop growth, development, and output and downregulate the overall socio-economic growth of sustainable agriculture. Different application strategies have been developed to challenge stress, its benefits, and its applications. Nowadays, the requirement for higher food grain productivity and safety, enhanced plant yield, fertility of soil properties, and agricultural sustainability are upregulating. The research demonstrations are shifting toward soil rhizospheric-bio-based engineering to facilitate a better pollution-free environment for combining plants and rhizobacteria. The application of PRs is more beneficial in overcoming stressed conditions besides providing other significant direct and indirect ways to upregulate overall plant responses. PRs are more convenient, economical, and eco-enviro-friendly and can be applied in small cultivating areas to large fields. Variations in the modifications of plant responses under stress have been observed in inoculated plants, and these variations are dependent on the PRs mode of action, which represents the multifactorial processes regulated in stressful environments. The positive symbiotic associanship that plants develop with microbial physiology is fundamental for the plant development, especially in terms of biotic and abiotic stresses. It is necessary to set up deeply extensive field research demonstrations to understand better the interaction between the PRs-mediated signal and the metabolic/molecular reprogramming that improves plant tolerance to unfavorable environmental variables. Multi-strain bacterial strains can be substantial if a single strain of bacteria is not more significant in reducing stress resistance efficiency. The application, duration, and applicability of inoculation are more crucial as unmanaged methods may lead to consistent and correct results. Its successful agro-commercialization will based on the involvement of plant physiologists, plant biologists, plant pathologists, biotechnologists, agro-industrialists, and farmers. A better and deep understanding of the action mechanisms and interactions of plants and associated plant rhizobacteria directly in the matrix of interest can be favored by the adoption of a holistic approach that uses “omic” applications.

## Author contributions

KV: Conceptualization, Data curation, Formal analysis, Methodology, Resources, Software, Validation, Writing – original draft. AJ: Conceptualization, Data curation, Formal analysis, Software, Writing – original draft. X-PS: Conceptualization, Data curation, Funding acquisition, Investigation, Project administration, Software, Supervision, Validation, Visualization, Writing – review & editing. QL: Data curation, Formal analysis, Funding acquisition, Resources, Software, Writing – review & editing. LX: Data curation, Formal analysis, Resources, Software, Writing – review & editing. H-rH: Data curation, Formal analysis, Resources, Software, Writing – review & editing. K-CW: Data curation, Funding acquisition, Resources, Software, Supervision, Writing – review & editing. CS: Data curation, Formal analysis, Resources, Software, Writing – review & editing. JA: Data curation, Formal analysis, Resources, Software, Writing – review & editing. Y-RL: Conceptualization, Funding acquisition, Investigation, Methodology, Project administration, Resources, Supervision, Validation, Visualization, Writing – review & editing.
